# Stereoscopic processing of crossed and uncrossed disparities in the human visual cortex

**DOI:** 10.1186/s12868-017-0395-7

**Published:** 2017-12-21

**Authors:** Yuan Li, Chuncheng Zhang, Chunping Hou, Li Yao, Jiacai Zhang, Zhiying Long

**Affiliations:** 1 School of Electrical and Information Engineering, Tianjin University, Tianjin, China; 2 State Key Laboratory of Cognitive Neuroscience and Learning, Beijing Normal University, Beijing, China; 3 College of Information Science and Technology, Beijing Normal University, Beijing, China

**Keywords:** fMRI, Crossed disparity, Uncrossed disparity, MVPA, Stereopsis

## Abstract

**Background:**

Binocular disparity provides a powerful cue for depth perception in a stereoscopic environment. Despite increasing knowledge of the cortical areas that process disparity from neuroimaging studies, the neural mechanism underlying disparity sign processing [crossed disparity (CD)/uncrossed disparity (UD)] is still poorly understood. In the present study, functional magnetic resonance imaging (fMRI) was used to explore different neural features that are relevant to disparity-sign processing.

**Methods:**

We performed an fMRI experiment on 27 right-handed healthy human volunteers by using both general linear model (GLM) and multi-voxel pattern analysis (MVPA) methods. First, GLM was used to determine the cortical areas that displayed different responses to different disparity signs. Second, MVPA was used to determine how the cortical areas discriminate different disparity signs.

**Results:**

The GLM analysis results indicated that shapes with UD induced significantly stronger activity in the sub-region (LO) of the lateral occipital cortex (LOC) than those with CD. The results of MVPA based on region of interest indicated that areas V3d and V3A displayed higher accuracy in the discrimination of crossed and uncrossed disparities than LOC. The results of searchlight-based MVPA indicated that the dorsal visual cortex showed significantly higher prediction accuracy than the ventral visual cortex and the sub-region LO of LOC showed high accuracy in the discrimination of crossed and uncrossed disparities.

**Conclusions:**

The results may suggest the dorsal visual areas are more discriminative to the disparity signs than the ventral visual areas although they are not sensitive to the disparity sign processing. Moreover, the LO in the ventral visual cortex is relevant to the recognition of shapes with different disparity signs and discriminative to the disparity sign.

## Background

Depth perception, which arises from a variety of depth cues, is an important visual ability for 3D perception. Binocular disparity is one of the powerful depth cues and is provided by the differences between the retinal images of the two eyes [1]. The brain uses binocular disparity to extract depth information from the two-dimensional retinal images in stereopsis. Investigation of the neural mechanism underlying binocular disparity can provide insights into the understanding of neural representations mediating depth perception.

Neurons selective for binocular disparity were first described in the V1 area of the cat [2–4]. Single-unit studies in macaques identified neurons that are sensitive to binocular disparity in many cortical areas, including the V1, V2, V3, V4, VP, MT, and MST areas [5–9]. Most studies of the human visual cortex using functional magnetic resonance imaging (fMRI) reported that the dorsal visual cortical areas V3A and V7 produced disparity-evoked responses [10, 11]. Moreover, other studies reported that the lateral occipital cortex, IPS, hMT+/V5, V3B, V4v, V8, etc., also responded to binocular disparity [12–14].

Although the regions that are engaged in binocular disparity have been studied extensively, only a few studies have investigated the brain regions that can discriminate different disparity signs [crossed disparity (CD)/uncrossed disparity (UD)]. Gilaie-Dotan et al. [15] compared the signal levels between “front” objects/gratings (UD) and “back” objects/gratings (CD). A significant preference to “front” objects over “back” objects was found in the lateral occipital complex (LOC) and dorsal foci (DF), which included the intraparietal sulcus and a region that overlaps area V3A [15]. Preston et al. [16] investigated multivoxel pattern selectivity for perceptually relevant binocular disparities in the human brain using multivoxel-pattern analysis (MVPA). Their study revealed that dorsal areas show a high discriminative power for metric disparity structure (i.e., disparity magnitude irrelevant to disparity sign), while the ventral lateral occipital area encodes depth position in a categorical manner (i.e., disparity sign). Patten et al. [17] used MVPA to decode depth positions (near vs. far) from fMRI activity and found that the V3d, V3A, LO and posterior parietal cortex showed high prediction accuracy above chance level. Moreover, V3A was observed to show the highest accuracies for discriminating cross (near) versus uncross (far) disparity [18]. Nasr et al. [19] conducted 7T fMRI measurements in human subjects during presentation of visual stimuli in near versus far conditions and found that ‘far—near’ binocular disparity contrast evoked activity in the portions of V2 and V3 that represent upper versus lower visual fields (i.e. ventral versus dorsal visual cortex respectively). Those previous studies demonstrated that both the ventral and dorsal visual cortex contributed to the disparity sign processing. However, some studies focused on dorsal visual areas while the others did on the ventral visual areas due to some restrictions of fMRI experiments such as limited scanning time and/or regions to be covered with an ultra-high field scan. Moreover, the function of the ventral and dorsal visual areas in the disparity sign processing is still not very clear. Therefore, it is essential to investigate the neural correlates of the disparity sign processing in both the ventral and dorsal visual areas.

The present study aimed to explore the neural mechanism mediating disparity-sign processing in the whole visual cortex using fMRI. Because large disparities can induce stronger neural activities in visual cortex than small disparities [20], a fixed large disparity was adopted in this study to avoid the interaction of the disparity magnitude and disparity sign. The general linear model (GLM) and MVPA methods were used in the present study to reveal different neural features underlying disparity-sign processing. First, we applied the general linear model (GLM) to investigate the neural differences of neural response between CD and UD [21]. Then, we applied MVPA method to obtain information on disparity sign discrimination that is represented in multivoxel patterns of activity [22]. We found that the dorsal areas, especially V3d and V3A, showed higher accuracy for discriminating shapes with different disparity signs than the ventral visual cortex. Moreover, the posterior subdivisions of the lateral occipital cortex (LOC) in the ventral visual cortex were more engaged in processing shapes with UD than CD and showed high accuracy in disparity sign discrimination.

## Methods

### Participants

We performed an fMRI experiment on 27 right-handed healthy human volunteers (12 male volunteers; mean age, 22.67 ± 2.96 years). All participants had normal or corrected-to-normal vision and were screened for stereo deficits using a stereo test. The stereo test guaranteed that the volunteers were able to distinguish between crossed and uncrossed disparities. The experiment was approved by the ethics committee of the Beijing Normal University. All participants provided written informed consent according to the guidelines of the MRI Center of Beijing Normal University.

### Data acquisition

Functional images were obtained using a 3-T Siemens scanner. Echo-planar imaging (EPI) and T1-weighted (1.33 × 1 × 1 mm) data were collected. The functional scans were acquired using EPI with the following parameters (echo time, TE = 30 ms, repetition time, TR = 2000 ms, flip angle = 90°, field of view, FOV = 200 × 200 mm, 3.13 × 3.13 mm in-plane resolution, 33 slices, slice thick = 3.5 mm and gap thick = 0.7 mm). The localizer scans were obtained using EPI with the following parameters (TE = 30 ms, TR = 2000 ms, flip angle = 90°; FOV = 192 × 192 mm, 3 × 3 mm in-plane resolution, 33 slices, slice thick = 3 mm and gap thick = 0 mm).

### Stimuli

**Fig. 1:**
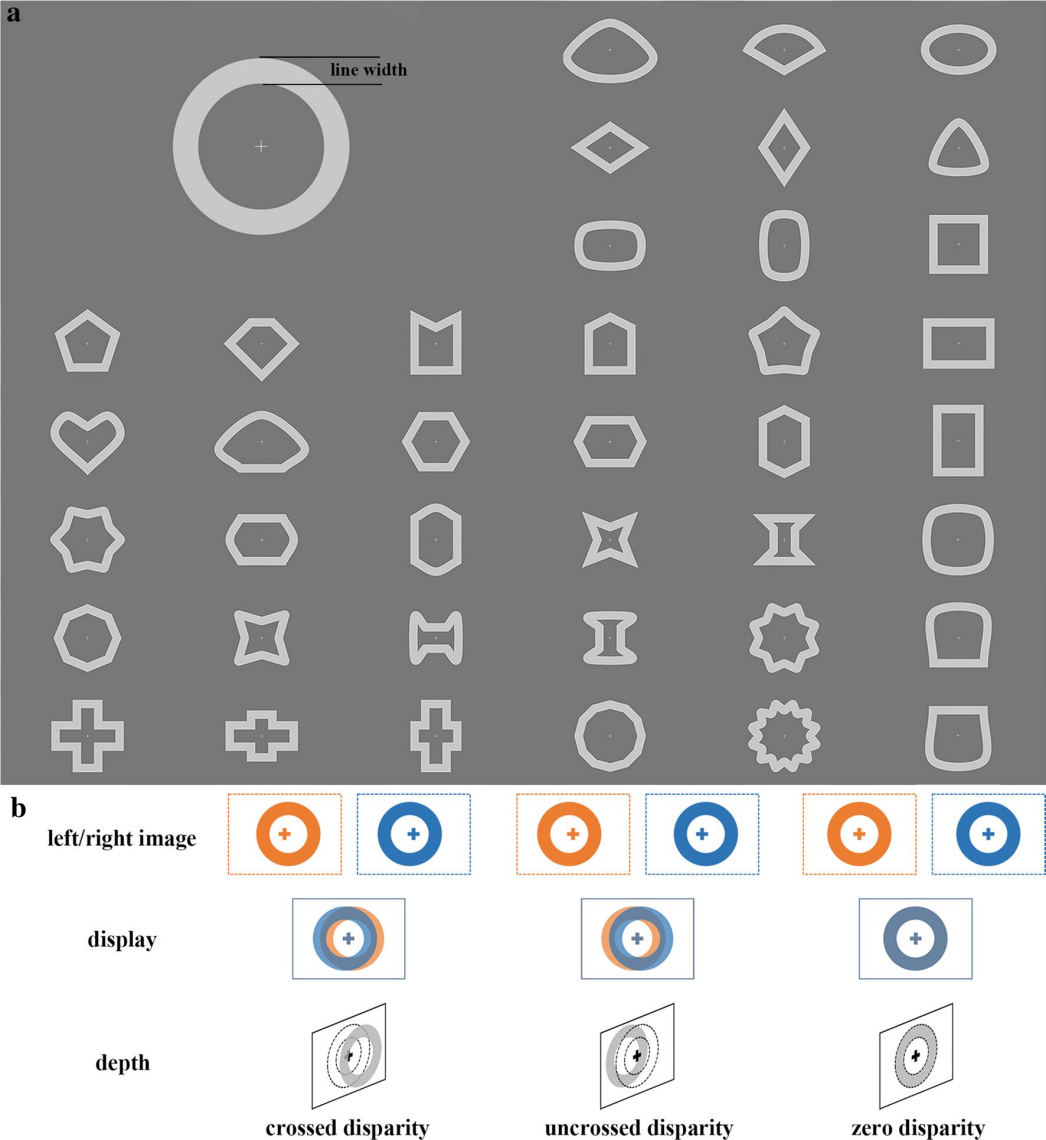
Schematic illustration of the stimuli. **a** Forty shapes that were used to generate stimuli with different disparities. **b** Diagram of the depth arrangement in the stimuli

Forty shapes (Fig. 1) with line width equal to 1.3° were used in the experiment. Each shape covered a maximum visual angle of 12° × 12° and was presented against a mid-gray background (26° × 14°). CIE 1931 xyY color space was used to describe the luminance and chromaticity values of the shape (Y = 1130, x = 1/3, y = 1/3) and background (Y = 672, x = 1/3, y = 1/3). For each shape, we generated three stimuli with 3 disparity levels (+ 30 arcmin, − 30 arcmin and 0 arcmin). Thus, the whole experiment consisted of 120 stimuli with different disparity levels. The positive disparity levels correspond to UD and the negative disparity levels correspond to CD. A cross fixation marker (0.6° × 0.6°) was presented in the center of the stimulus to assist in maintaining eye’s fixation. The stimuli were displayed using a 3D LCD with LED Backlight (LG D2343p, 1920 × 1080 pixels) positioned in the bore of the magnet at a distance of 110 cm from the point of observation. Participants wore polarized glasses and viewed the stimuli through a mirror tilted at 45°, which was attached to the head coil.

### Experimental design

The fMRI experiment used a block design and consisted of two runs. The three tasks that included CD, UD, and zero disparity (ZD) judgments involved the presentation of the sequences ABCBCACBACAB and BACACBCABCBA for the first and second runs, respectively. Each run consisted of twelve 24-s task blocks alternated with twelve 12-s fixation blocks. An individual trial lasted 2 s, comprising a stimulus presentation period of 1.5 s and an inter-stimuli interval of 0.5 s. Twelve trials with a specific disparity level were presented in a task block. For each task block, participants were required to press the button with the right index finger if the two continuous stimuli were different and the left index finger if the two continuous stimuli were the same. For the fixation blocks, participants were required to fixate on a cross at the center of the screen.

### Mapping visual regions of interest

For each participant, regions of interest (ROIs) were defined using standard mapping techniques. Retinotopic areas V1, V2d, V3d, V3A, V7, V2v, V3v, and V4v were defined using rotating wedges and expanding concentric rings stimuli [23]. V4v was defined as the region of retinotopic activation in the ventral visual cortex that was adjacent to V3v [24, 25]. V7 was defined as a region anterior and dorsal to V3A [11, 25, 26]. Furthermore, higher dorsal and ventral regions that included human motion complex (hMT+/V5) and the LOC were defined using independent localizer scans. Area hMT+/V5 was defined as the set of voxels in the lateral temporal cortex that responded with a significantly higher rate (*p* < 10^−4^) to a coherently moving array of dots than to a static array of dots [27]. The LOC was defined as the set of voxels in the lateral occipito-temporal cortex that produced significantly strong responses (*p* < 10^−3^) to intact than scrambled images of objects [28].

### ROI-based MVPA

#### Preprocessing

The disparity experiment data of each subject were analyzed using BrainVoyager QX (BrainInnovation). The preprocessing of each subject’s functional data included three-dimensional correction of head movements, slice scan time correction and temporal high-pass filtering (2 cycles per run cutoff). Anatomical scans were transformed into Talairach space data and used for 3D cortex reconstruction, inflation, flattening, and segmentation of gray and white matter. Functional imaging data were aligned using the anatomical data as a positional reference and transformed into Talairach space.

For the ROI localizer runs, the preprocessing of each subject’s functional data underwent the same preprocessing steps, except that anatomical scans were transformed into Talairach and Montreal Neurological Institute (MNI) space respectively. The functional data of the ROI localizer runs were aligned using the anatomical data as a positional reference and transformed into both Talairach and MNI space respectively. Thus, the ROIs in both Talaraich and MNI space can be defined.

#### Classification

For each visual ROI in Talaraich space, gray matter voxels were selected from both hemispheres and sorted according to their T-statistics that were calculated by contrasting all stimulus conditions (CD, UD, and ZD) to the fixation baseline across 2 runs. The same number of voxels was selected across ROIs and participants. For each hemisphere of each ROI, 100 voxels were selected from among those voxels with T > 0 for contrasting between “all stimuli and fixation.” For some cortical areas in some subjects, 200 voxels may not be available. In such cases, we used the maximum number of voxels that had a T-statistics greater than 0. For example, only 194 voxels were selected from area V3v in participant 1.

The time course of each voxel was detrended and transformed into a z-score for each experimental run of each subject. Moreover, the volume at each time point was also transformed into a z-score. The fMRI time series was shifted by 2 TRs to account for the hemodynamic delay of the BOLD signal. An eightfold leave-one-out cross-validation was performed for each participant’s data. The trials corresponding to UD, CD, and ZD from 2 runs were shuffled and divided into 8 parts. For each cross-validation, data from one part was discerned as an independent-test dataset and that from the remaining 7 parts were used as the training set. For each ROI, the selected voxels from the training dataset were used to estimate the support vector machine (SVM) classifier based on a linear kernel. SVM was implemented using LIBSVM software (www.csie.ntu.edu.tw/~cjlin/libsvm/) and the parameter c was set to 1. After the three two-class SVM classifiers (CD and UD, CD and ZD, and UD and ZD) were trained, they were applied to the testing data to classify the task state of each trial. The prediction accuracy of the classifier was determined by calculating the ratio of the number of the trials that were correctly classified to the total number total trials. For each participant, the mean prediction accuracy of each ROI was obtained by determining the average of the prediction accuracy across eightfold cross-validations. A one-way repeated measure ANOVA using the ROI as the within-subject factor was performed to evaluate the differences of the prediction accuracy rate among the 10 ROIs in SPSS v20. In addition, the further simple effect test was performed using a multiple comparison correction with a Sidak test.

To further examine the differences of prediction accuracy between the ventral visual area and the dorsal visual area, the ventral and dorsal ROIs were defined. The ventral ROI consisted of the voxels from V2v, V3v, V4v, and LOC in Talaraich space. The dorsal ROI consisted of the voxels from V2d, V3d, V3A, V7, and hMT+/V5 in Talaraich space. For each participant, the SVM training and test procedures described above were performed separately on the ventral and dorsal ROIs. The mean prediction accuracy of each ROI was calculated. A paired *t* test in SPSS v20 was performed to examine the differences in prediction accuracy between the ventral and dorsal ROIs.

To determine the chance level of classifier and judge the reliability of the results, the prediction analysis with randomly permuted fMRI patterns were applied to each ROI by randomizing the correspondence between the fMRI data and the training labels 1000 times for all the two-class classifications (CD versus UD, CD versus ZD, UD versus ZD). Thus, a distribution of prediction accuracies was created and the upper 99.5% centile was used as the chance level of classifier based on the distribution.

### Searchlight-based MVPA

#### Preprocessing

The disparity experiment data were corrected for slice acquisition timing and motion, spatially normalized to a standard stereotaxic space (MNI EPI template), and resampled to an isotropic spatial resolution of 3 × 3 × 3 mm^3^ in SPM8 software (http://www.fil.ion.ucl.ac.uk/spm).

#### Analysis of whole brain

The ROI-based MVPA has the advantage of independent localization of areas that are based on functional delineation. However, to determine whether some important information might be ignored or missed by using this approach, we conducted a searchlight-based classification analysis of whole brain [29]. In particular, we moved a small sphere ROI (radius = 9 mm, 33 voxels) sequentially through the whole brain and conducted the three two-class classification analyses (CD versus UD, CD versus ZD and UD versus ZD) to provide three prediction accuracy maps for each participant. The same classification analysis method as the ROI-based MVPA was used.

To perform group analysis, the prediction accuracy maps of each participant were first transformed into a z-score map. Then one-sample *t* test was performed to identify the brain regions that showed high prediction accuracy for CD versus UD, CD versus ZD and UD versus ZD separately. The results of statistical analysis were corrected using a topological FDR based on peak, with a threshold of *p* = 0.01 [30].

#### Ventral and dorsal comparison

To examine the prediction accuracy differences between the ventral and the dorsal visual ROIs for each two-class classification, the ventral and dorsal ROIs were defined. The ventral ROI consisted of the voxels from V2v, V3v, V4v, and LOC in MNI space. The dorsal ROI consisted of the voxels from V2d, V3d, V3A, V7, and hMT+/V5 in MNI space. For each participant, the mean prediction accuracy of each two-class classifications (CD versus UD, CD versus ZD and UD versus ZD) within the ventral/dorsal ROI was calculated. A paired *t* test in SPSS v20 was performed to examine the differences in prediction accuracy between the ventral and dorsal ROIs for each classification.

### GLM analysis

#### Preprocessing

The same preprocessing steps as the searchlight-based MVPA were performed. In addition, the disparity experiment data were spatially smoothed with an isotropic Gaussian kernel of 4 mm in SPM8 software.

#### Analysis of whole brain

The GLM analysis in SPM8 was applied to the functional images of each participant using CD, UD, and ZD as the three regressors. The group datasets were analyzed using a random effects model. A one-way within-subject ANOVA was performed to identify the brain regions that showed significant differences for CD > ZD, UD > ZD, CD > UD, and UD > CD. The results of statistical analysis were corrected using a topological false discovery rate (FDR) based on clusters, with a cluster-defining threshold of *p* = 0.001 [30].

The results of the group analysis were mapped onto the human Population-Averaged, Landmark- and Surface-based (PALS) atlas surface in MNI space using the Caret software package [31, 32]. Caret software and the PALS atlas are available at http://brainvis.wustl.edu/wiki/index.php/Caret:Download and http://sumsdb.wustl.edu/sums/directory.do?id=636032, respectively.

### Ventral and dorsal comparison

To identify whether there were significant response differences for CD > ZD, UD > ZD, CD > UD, and UD > CD in the ventral and the dorsal visual area. For the ventral/dorsal ROI of each participant in MNI space, the contrast value of each contrast (CD > ZD, UD > ZD, CD > UD, and UD > CD) was averaged across the voxels within ROI. Then, mean contrast value of each ROI was calculated across the participants. A paired *t* test in SPSS v20 was performed to examine the differences between the ventral and dorsal ROIs.

## Results

### ROI-based MVPA

**Fig. 2:**
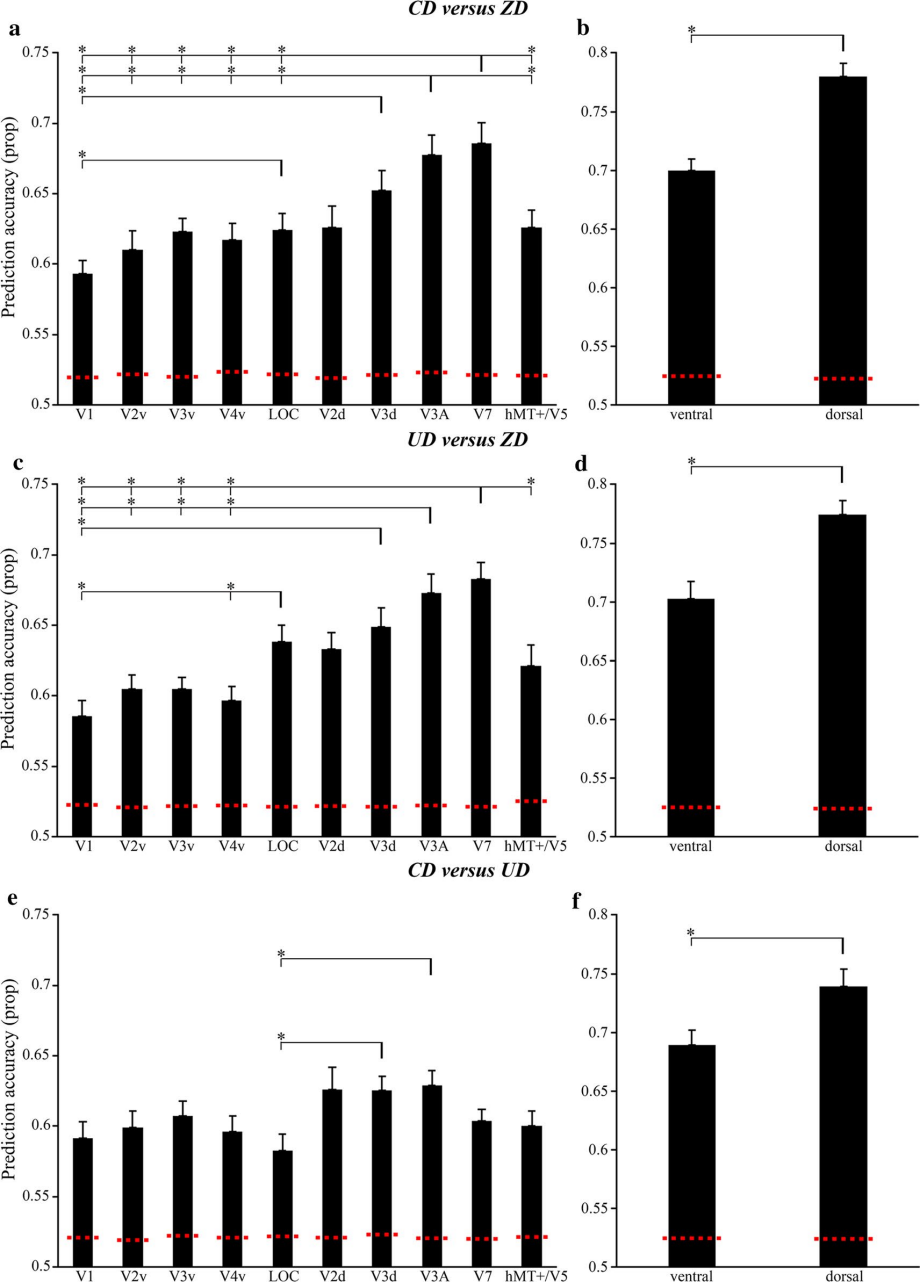
Prediction accuracy of MVPA based on ROI. **a** The mean prediction accuracy of CD versus ZD for ten ROIs. **b** The mean prediction accuracy of CD versus ZD for ventral and dorsal ROIs. **c** The mean prediction accuracy of UD versus ZD for ten ROIs. **d** The mean prediction accuracy of UD versus ZD for ventral and dorsal ROIs. **e** The mean prediction accuracy of CD versus UD for ten ROIs. **f** The mean prediction accuracy of CD versus UD for ventral and dorsal ROIs. The error bars represent the standard error of mean. * *p* < 0.05. The dotted horizontal red lines mark the chance level generated from permuting the data labels before being fed into the classifier. The location of the line indicates the upper 99.5% centile of the distribution of permuted data

Figure 2a, b shows the mean prediction accuracies of each ROI for discriminating between CD and ZD. The mean prediction accuracy of all ROIs was above chance levels (see Fig. 2a, b, red dotted lines). Moreover, areas V3d, V3A, and V7 showed prediction accuracy higher than 0.65 and LOC showed prediction accuracy higher than other ventral areas. A one-way ANOVA of 10 ROIs revealed a significant ROI effect (*F*_(9,234)_ = 8.962; *p* < 0.0005). Further simple effect analysis showed that area V3d had significantly higher prediction accuracy than that of area V1. The prediction accuracy of area V3A was significantly higher than that of areas V1, V2v, V3v, V4v, LOC and hMT+/V5. The prediction accuracy of area V7 was significantly higher than that of areas V1, V2v, V3v, V4v, LOC and hMT+/V5. The prediction accuracy of area LOC was significantly higher than that of area V1. Table 1 presents *p*-values for the simple-effect analysis. Furthermore, the paired-sample *t* test of ventral and dorsal ROIs also revealed that the dorsal ROI had a significantly higher prediction accuracy than the ventral ROI (T_(26)_ = 7.27, *p* < 0.0005).

**Table 1:** Statistical comparisons of prediction accuracy for CD versus ZD

	V1	V2v	V3v	V4v	LOC	V2d	V3d	V3A	V7	hMT+/V5
V1	–	1.000	0.157	0.977	0.908	0.878	0.046*	< 0.0005*	< 0.0005*	0.612
V2v	–	–	1.000	1.000	1.000	1.000	0.233	0.012*	0.002*	1.000
V3v	–	–	–	–	1.000	1.000	0.822	0.007*	0.001*	1.000
V4v	–	–	1.000	–	1.000	1.000	0.595	0.019	0.005*	1.000
LOC	–	–	–	–	–	1.000	0.750	0.025*	0.010*	1.000
V2d	–	–	–	–	–	–	0.976	0.163	0.096	–
V3d	–	–	–	–	–	–	–	0.995	0.826	–
V3A	–	–	–	–	–	–	–	–	1.000	–
V7	–	–	–	–	–	–	–	–	–	–
hMT+/V5	–	–	–	–	0.577	1.000	0.980	0.007*	0.004*	–

This table provides *p*-values for all pairwise ROI comparisons (p-values rounded up). The symbol ‘*’ indicates that the ROI in the corresponding column showed a significantly higher prediction accuracy than the ROI in the corresponding row (*p* < 0.05). The symbol ‘–’ indicates that prediction accuracy for the column ROI was less than or equal to that for the row ROI

Figure 2c, d shows the mean prediction accuracy of each ROI for discriminating between UD and ZD. The mean prediction accuracy of all ROIs was above chance levels (see Fig. 2c, d, red dotted lines). Moreover, areas V3A and V7 showed a prediction accuracy higher than 0.65 and LOC showed a prediction accuracy higher than other ventral areas. A one-way ANOVA of 10 ROIs revealed a significant ROI effect (*F*_(9,234)_ = 11.277; *p* < 0.0005). Further simple effect analysis showed that area V3d had significantly higher prediction accuracy than that of area V1. The prediction accuracy of area V3A was significantly higher than that of areas V1, V2v, V3v and V4v. The prediction accuracy of area V7 was significantly higher than that of areas V1, V2v, V3v, V4v and hMT+/V5. The prediction accuracy of area LOC was significantly higher than that of areas V1 and V4v. Table 2 provides *p*-values for all of the simple-effect analysis. Furthermore, the paired-sample *t* test of ventral and dorsal ROIs revealed that the dorsal ROI showed a significantly higher prediction accuracy than the ventral ROI (T_(26)_ = 6.37, *p* < 0.0005).

**Table 2:** Statistical comparisons of prediction accuracy for UD versus ZD

	V1	V2v	V3v	V4v	LOC	V2d	V3d	V3A	V7	hMT+/V5
V1	–	0.983	1.000	1.000	< 0.0005*	0.085	0.009*	< 0.0005*	< 0.0005*	0.787
V2v	–	–	1.000	–	0.154	0.630	0.129	0.003*	< 0.0005*	1.000
V3v	–	–	–	–	0.042*	0.756	0.213	0.001*	< 0.0005*	1.000
V4v	–	1.000	1.000	–	0.009*	0.352	0.116	0.006*	< 0.0005*	0.986
LOC	–	–	–	–	–	1.000	1.000	0.703	0.062	1.000
V2d	–	–	–	–	–	–	1.000	0.072	0.145	–
V3d	–	–	–	–	–	–	–	0.980	0.866	–
V3A	–	–	–	–	–	–	–	–	1.000	–
V7	–	–	–	–	–	–	–	–	–	–
hMT+/V5	–	–	–	–	0.484	1.000	1.000	0.072	0.006*	–

This table provides *p*-values for all pairwise ROI comparisons (*p*-values rounded up). The symbol ‘*’ indicates that the ROI in the corresponding column showed a significantly higher prediction accuracy than the ROI in the corresponding row (*p* < 0.05). The symbol ‘–’ indicates that prediction accuracy for the column ROI was less than or equal to that for the row ROI

Figure 2e, f shows the mean prediction accuracies of each ROI for discriminating between CD and UD. The mean prediction accuracy of all the ROIs was above chance levels (see Fig. 2e, f, red dotted lines). Moreover, areas V2d, V3d, and V3A showed higher prediction accuracy than the others while LOC showed lowest prediction accuracy than the others. A one-way ANOVA of 10 ROIs revealed a significant ROI effect (*F*_(9,234)_ = 3.000; *p* < 0.005). Further simple-effect analysis showed that area V3d and V3A had a significantly higher prediction accuracy than that of areas LOC. Table 3 lists the *p*-values for all the simple effect analysis tests. Furthermore, the paired-sample *t* test of ventral and dorsal ROIs also revealed that the dorsal ROI showed a significantly higher prediction accuracy than the ventral ROI (T_(26)_ = 3.87, *p* < 0.001).

**Table 3:** Statistical comparisons of prediction accuracy for CD versus UD

	V1	V2v	V3v	V4v	LOC	V2d	V3d	V3A	V7	hMT+/V5
V1	–	1.000	1.000	1.000	–	0.966	0.627	0.398	1.000	1.000
V2v	–	–	1.000	–	–	0.988	0.811	0.712	1.000	1.000
V3v	–	–	–	–	–	1.000	1.000	0.992	–	–
V4v	–	1.000	1.000	–	–	0.727	0.673	0.410	1.000	1.000
LOC	1.000	1.000	0.773	1.000	–	0.246	0.033*	0.009*	0.799	0.989
V2d	–	–	–	–	–	–	–	1.000	–	–
V3d	–	–	–	–	–	1.000	–	1.000	–	–
V3A	–	–	–	–	–	–	–	–	–	–
V7	–	0.434	1.000	–	–	1.000	0.934	0.589	–	–
hMT+/V5	–	0.343	1.000	–	–	0.989	0.766	0.407	1.000	–

This table provides p-values for all pairwise ROI comparisons (p-values rounded up). The symbol ‘*’ indicates that the ROI in the corresponding column showed a significantly higher prediction accuracy than the ROI in the corresponding row (*p* < 0.05). The symbol ‘–’ indicates that prediction accuracy for the column ROI was less than or equal to that for the row ROI

### Searchlight-based MVPA

#### Analysis of whole brain

**Fig. 3:**
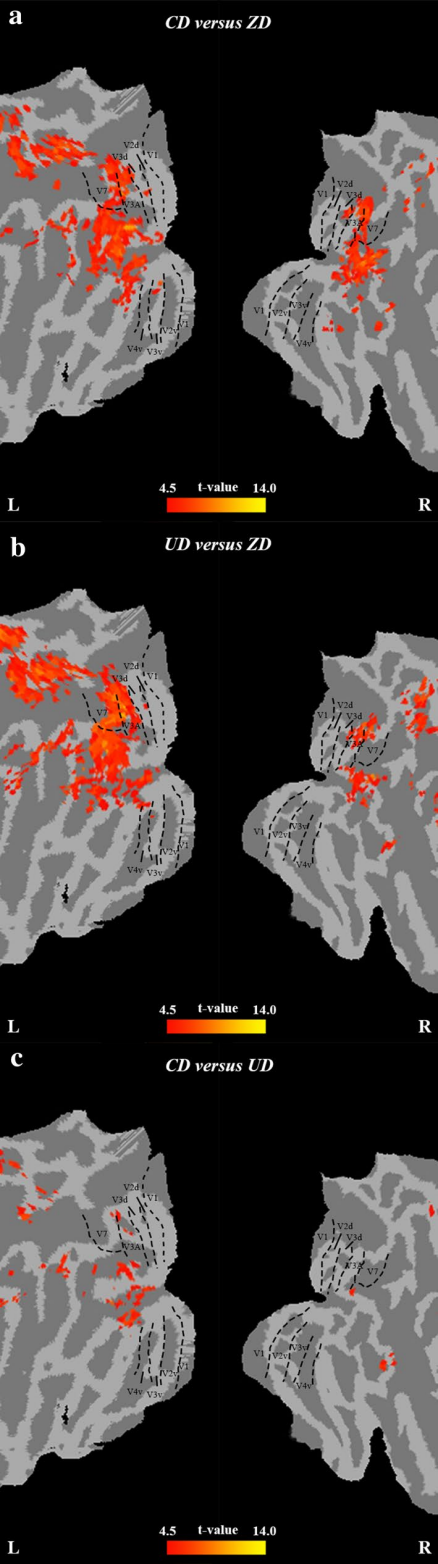
Statistical parametric maps projected onto the flattened cortical representations for the searchlight analysis. Brain areas that show significantly high prediction accuracy for CD versus ZD (**a**) UD versus ZD (**b**) and CD versus UD (**c**). The statistical results were corrected using a topological false discovery rate (FDR) based on peak with a threshold of *p* = 0.01. The black dashed lines described the retinotopic areas V1, V2d, V3d, V3A, V7, V2v, V3v, and V4v (from Caret atlas)

Figure 3 shows the regions with significant higher prediction accuracies for the discrimination of CD versus ZD, UD versus ZD and CD versus UD. For the classifications of CD versus ZD and UD versus ZD, voxels with high accuracies located in bilateral V3A, V3B, V7, IPS, hMT+/V5, LO1, LO2 and MTG according to the coordinates in the previous studies [13, 33–36]. For the classification of CD versus UD, most voxels with high accuracies located in left V3A, left V3B, left IPS, left LO1 and left LO2, and others located in left V7, right V3B, right IPS and bilateral MTG according to the coordinates in the previous studies [13, 33–35].

#### Ventral and dorsal comparison

**Fig. 4:**
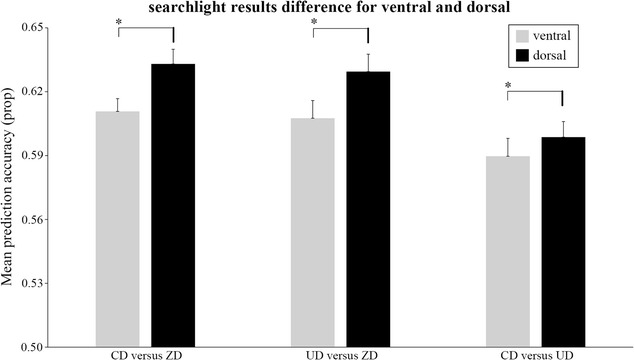
The mean searchlight statistic values of the ventral and dorsal ROIs for CD versus ZD, UD versus ZD and UD versus CD. The error bars represent the standard error of mean. * *p* < 0.05

Figure 4 shows the mean prediction accuracies of the ventral and dorsal ROIs for the three two-class classifications (CD versus ZD, UD versus ZD and UD versus CD). The paired-sample *t* tests revealed that the mean prediction accuracy of the dorsal ROI was significantly higher than that of the ventral ROI for CD versus ZD (T_(26)_ = 5.744, *p* < 0.0005), UD versus ZD (T_(26)_ = 5.300, *p* < 0.0005) and CD versus UD (T_(26)_ = 2.802, *p* < 0.01).

### GLM results

#### Analysis of whole brain

**Fig. 5:**
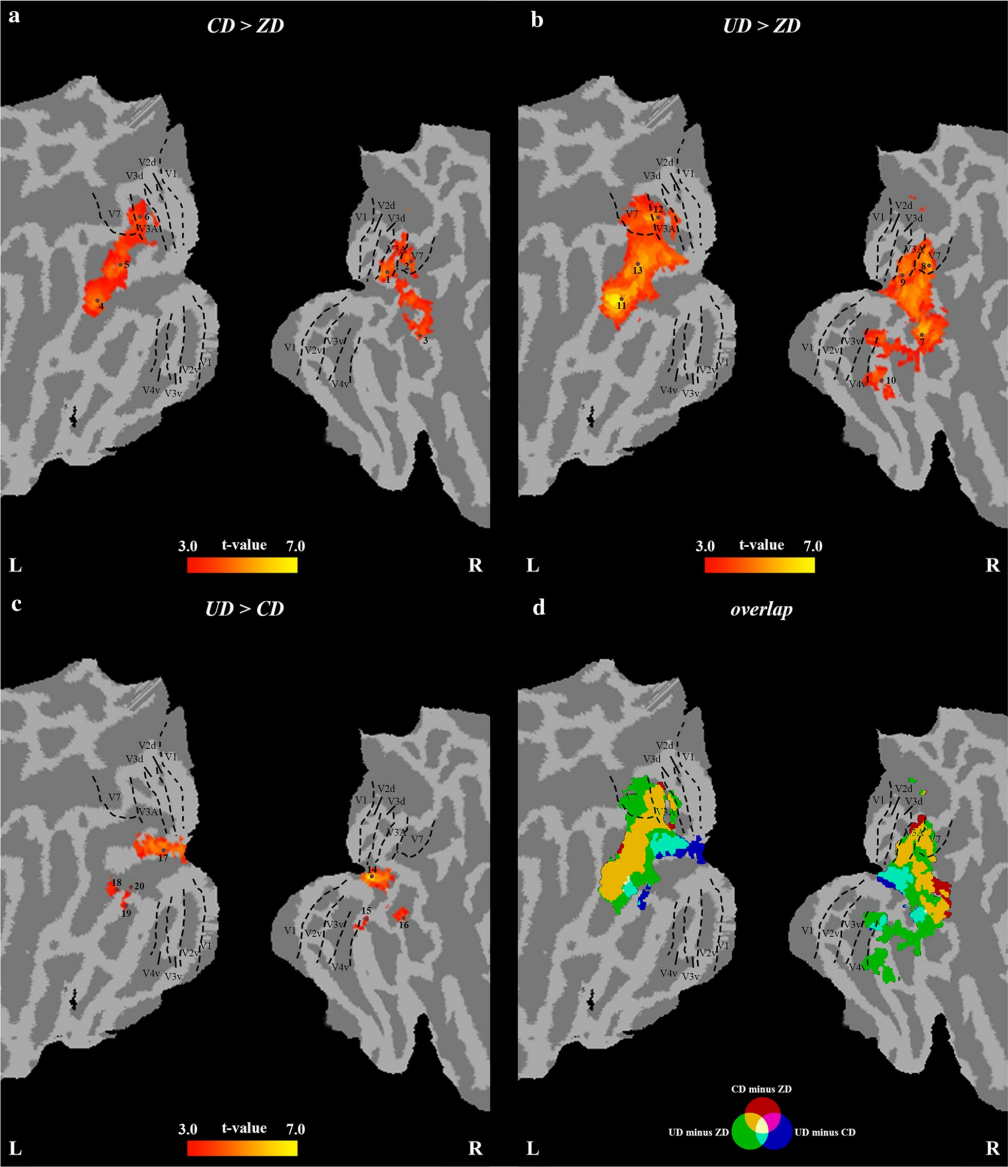
Statistical parametric maps projected onto the flattened cortical representations for the GLM analysis. Brain regions that show significantly stronger responses for CD > ZD (**a**), UD > ZD (**b**) and UD > CD (**c**). **d** The overlapping map of the results in **a–c**. The statistical results were corrected using a topological false discovery rate (FDR) based on clusters with a cluster-defining threshold of *p* = 0.001. The significant local maxima voxels are indicated as numbers and brown points

Figure 5a–c shows the regions with significant difference for CD > ZD, UD > ZD, and UD > CD. The brain regions that showed significant differences for CD > UD were not found. The results are displayed on the rendered cortex and in the flat map of the PALS atlas. The statistical local maxima are indicated using brown dots and numbers in Fig. 5a–c, and correspond to the coordinates in Table 4. The coordinates of local maxima in MNI space and in Talairach space [37] are summarized in Table 4.

**Table 4:** Activated brain regions for CD > ZD, UD > ZD and UD > CD

	Index	MNI coordinates			Talairach coordinates			T-score	Area
		x	y	z	x	y	z		
CD > ZD	1	24	− 91	22	24	− 87	25	4.96	V3A
	2	27	− 76	34	27	− 72	35	4.74	V7
	3	45	− 67	3	45	− 65	6	4.26	hMT+/V5
	4	− 45	− 73	4	− 45	− 71	7	5.08	LO2
	5	− 36	− 85	13	− 36	− 82	16	4.68	LO1
	6	− 24	− 85	28	− 24	− 81	30	4.51	V3A
UD > ZD	7	48	− 73	− 5	48	− 71	− 1	5.87	LO2
	8	30	− 79	28	30	− 75	30	5.68	V7
	9	24	− 91	19	24	− 87	22	4.96	V3A
	10	30	− 46	− 17	30	− 45	− 12	4.35	V8
	11	− 45	− 76	4	− 45	− 73	7	6.92	LO2
	12	− 24	− 86	28	− 24	− 82	30	5.59	V3A
	13	− 36	− 85	13	− 36	− 82	16	5.74	LO1
UD > CD	14	31	− 94	4	31	− 91	8	6.71	LO1
	15	39	− 83	− 8	39	− 81	− 3	3.94	LO1
	16	48	− 73	− 5	48	− 71	− 1	3.91	LO2
	17	− 21	− 94	4	− 21	− 91	8	5.93	LO1
	18	− 42	− 76	− 2	− 42	− 74	2	4.93	LO2
	19	− 36	− 77	− 11	− 36	− 75	− 5	3.92	LO2
	20	− 39	− 85	− 5	− 39	− 83	0	3.67	LO2

MNI coordinates, Talairach coordinates, T-score and anatomical area of local maxima were listed

For CD > ZD, significantly higher activation was found in the dorsal and lateral occipital areas (Fig. 5a). Site 1 was located in the right V3A area according to the coordinates proposed by Kujovic et al. [35]. Site 2 was located in the right V7 area according to the study of Press et al. [33]. Site 3 in the right middle temporal gyrus (MTG) might correspond to the hMT+/V5 area according to the coordinates proposed by Malikovic [36]. Site 4 in the left middle occipital gyrus (MOG) might correspond to the left lateral occipital areas 2 (LO2) based on the coordinates proposed by Malikovic [34]. Site 5 in the left middle occipital gyrus (MOG) might correspond to the left lateral occipital areas 1 (LO1) based on the coordinates proposed by Malikovic [34]. Site 6 was located in the right V3A area according to the coordinates proposed by Kujovic [35].

For UD > ZD, significantly higher activation was also found in the dorsal areas and ventral areas (Fig. 5b). Site 7 in the right inferior temporal gyrus (ITG) might correspond to the right lateral occipital areas 2 (LO2) based on the coordinates proposed by Malikovic [34]. Site 8 was located in the right V7 area according to the study of Press [33]. One pair of local maxima (sites 9 and 12) were located in the V3A area according to the coordinates proposed by Kujovic [35]. Site 10 was located in the right V8 area according to the study of Hadjikhani [38]. Two sites (11 and 13) in the left middle occipital gyrus (MOG) might correspond to the left LO2 and LO1 based on the coordinates proposed by Malikovic [34].

For UD > CD, lateral occipital areas showed significantly higher activation (Fig. 5c). One pair of local maxima (sites 14 and 17) in the right and left MOG and site 15 in the right inferior occipital gyrus (IOG) might correspond to LO1 according to the coordinates of Malikovic [34]. Site 16 in the right ITG and site 18 in the left MOG might correspond to LO2 according to the coordinates of Malikovic [34]. Two sites (19 and 20) in the left IOG might correspond to the left LO2 based on the coordinates proposed by Malikovic [34].

Figure 5d shows the overlapping map of the results for CD > ZD and UD > ZD and UD > CD. The regions showed higher activation for CD > ZD, UD > ZD and UD > CD were color coded in red, yellow and blue respectively. The regions responding preferentially to disparity information (CD/UD) was color coded in orange and covered areas V3A, V3B, V7, LO2 and hMT+/V5. The regions responding preferentially to UD information was color coded in celeste and covered areas LO1 and LO2.

### Ventral and dorsal comparison

**Fig. 6:**
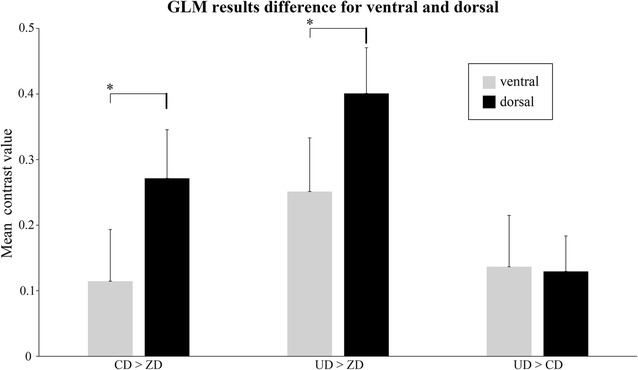
The mean contrast values of ventral and dorsal ROIs for CD > ZD, UD > ZD and UD > CD. The error bars represent the standard error of mean. * *p* < 0.05

Figure 6 shows the mean contrast values for each contrast in the ventral and dorsal ROIs. It can be seen that the mean contrast values within the dorsal ROI was significantly greater than the ventral ROI for CD > ZD (T_(26)_ = 3.328, *p* < 0.005) and UD > ZD (T_(26)_ = 3.437, *p* < 0.005). For UD > CD, no significant differences (T_(26)_ = 0.161, *p* > 0.05) between ventral and dorsal ROIs were observed.

## Discussion

In the present study, we applied GLM and MVPA methods to investigate the neural mechanism relevant to binocular disparity-sign processing. Firstly, MVPA results revealed that the dorsal visual cortex showed significantly higher accuracy to for discriminating UD versus CD, CD versus ZD and UD versus ZD in contrast to the ventral visual cortex. Secondly, GLM and MVPA results revealed that the subregion LO1 and LO2 in ventral visual area produced stronger responses to UD than CD and showed high prediction accuracy to discriminate UD and CD. Thirdly, the GLM results revealed that the dorsal visual cortex produced significantly stronger activities in disparity processing than the ventral visual cortex.

### Neural mechanism underlying disparity processing

Both GLM and MVPA analyses revealed that dorsal visual areas V3A, V3B and V7 produced significantly stronger activation for UD/CD compared to ZD, and showed a significantly high discriminative power for discriminating UD/CD and ZD (see Figs. 2a, c, 3a, b, 4, 5a, b, d, 6). In contrast to the ventral visual cortex, the dorsal visual cortex showed significantly higher responses for UD/CD compared to ZD and significantly higher accuracy for the discrimination of UD/CD and ZD (see Figs. 2b, d, 4, 6). Previous studies demonstrated that the comparatively earlier stages in the dorsal stream showed the selectivity for perceived depth [16] and dorsal medial visual cortex showed strong responses to stimuli with disparity in human participants [10, 11]. Our results provided converging evidence to further support that the dorsal visual areas, especially V3A, V3B and V7, responded preferentially to the disparity information and had higher disparity discrimination power in contrast to the ventral visual cortex.

Within the ventral visual cortex, the LO1 and LO2 in LOC showed selective responses to the shapes with disparities and high accuracies in discriminating the shapes with and without disparities (see Figs. 3a, b, 5a, b). Although the early and intermediate ventral visual cortex, such as V1,V2 and V4v, were reported to be engaged in the disparity processing [12, 19], they did not show strong responses to shapes with disparities in this study. In contrast, this study found that the higher ventral area LOC was relevant to the disparity perception. The stimuli used in those previous studies were random-dot stereograms rather than the shapes with disparities and LOC is reported to be relevant to shape processing [28, 39], which possibly resulted in lack of the activation of early and intermediate ventral visual cortex in this study. Macaque electrophysiological studies pointed out that neurons near the final stage of the ventral visual pathway, area TE, responds to stimuli that were defined by horizontal disparity [40–42]. Because the macaque area TE generally corresponds to LOC in humans [39, 43] and the horizontal disparity was used to generate 3D shapes in this study, it is reasonable that LOC produced high activation in processing the shapes with disparities and showed high disparity discrimination power. It can be inferred that the LOC may use the disparity information predominantly to facilitate shape recognition.

### Neural mechanism underlying disparity sign processing

In the dorsal visual pathway, the searchlight-based MVPA revealed that left V3A, bilateral V3B, left V7 and left IPS showed high discriminative power (see Fig. 3c) and ROI-based MVPA revealed that V3d and V3A showed significantly higher accuracy than LOC for the discrimination of UD and CD (see Fig. 2e). Moreover, both searchlight-based and ROI-based MVPA found that the dorsal visual cortex showed significantly higher accuracy than the ventral visual cortex (see Figs. 2f, 4). Previous studies also reported that V3A, V3B, V3d and V7 had high accuracy in the classification of UD and CD [16–18], which are consistent with the present study. Our results may suggest that the dorsal pathway might contain some useful discrimination information of disparity sign. However, the GLM analysis did not detect the significant differences between UD and CD in the dorsal visual cortex. Previous study demonstrated that the activities of the dorsal visual cortex increased with the disparity magnitude [20]. In this study, the disparity magnitude of UD was the same as that of CD. The results that the dorsal visual regions were engaged in disparity processing but showed similar activation between UD and CD could further suggest that the dorsal pathway was relevant to the disparity magnitude processing rather than the disparity sign processing.

In the ventral pathway, serachlight-based MVPA found that LO1 and LO2 in LOC had high accuracy for the discrimination of UD and CD (see Fig. 3c). However, the ROI-based MVPA revealed that LOC showed the lowest accuracy among all the ROIs for the discrimination of UD and CD (see Fig. 2e). Some studies suggested that LOC was likely to be a complex of several subdivisions and could include LO (LO1 and LO2) and posterior fusiform (pFs) [44, 45]. The LOC regions that were revealed by searchlight-based MVPA mainly located in LO while the LOC ROI that was defined in this study included LO and pFS. Because our results did not found that pFS in LOC was relevant to the disparity processing, the low accuracy of the LOC ROI for discriminating UD versus CD may be attributed to the inclusion of pFS in LOC. Previous monkey physiological studies pointed out that the lower bank of the superior temporal sulcus rather than the lateral TE was a possible candidate for selective processing of 3D object structure [46]. The lower bank of the superior temporal sulcus corresponds to caudal-dorsal subdivision of the LOC (LO) and the lateral TE corresponds to ventral-anterior part of the LOC (pFS) [39]. The high accuracy of LO for the classifications of UD versus CD possibly suggested that LO was discriminative to different disparity signs. Previous study also demonstrated that LO appeared to represent depth position in a categorical manner (i.e., disparity sign) [16]. Therefore, our results provided evidence to further support that the function of LO and pFS in LOC may be segregated.

Results of GLM further showed that LO (LO1 and LO2) instead of pFS in LOC produced significantly stronger responses to UD compared to CD (see Fig. 5c). It should be noted that there may exist the perceived size effect that the distant shapes are perceived larger compared to close shapes if the retinal sizes of objects are the same [47, 48]. In this study, all the stimuli had the same physical size. The retinal size differences between UD and CD shapes were too small to be noticed because the focus distance was much larger than the size of shapes in our experiment. Due to the similar retinal size of UD and CD, the UD shapes were perceived larger in contrast to CD shapes in this study. Previous studies demonstrated that LO was sensitive to the size changes while pFs was not [44, 49]. Moreover, larger stimuli tend to induce stronger brain activities than smaller stimuli. Accordingly, it was reasonable that LO instead of pFS produced greater responses to UD shapes than CD shapes in this study. Murray et al. [47] demonstrated that the effect of perceived size differences was clearly reflected in the V1 and indicated that the feedback from higher visual areas would seem to make an important contribution to the effect. Although the perceived size differences were not observed in V1 in this study, our results suggested the feedback of LO in the higher visual areas might contribute to the size effect. However, the GLM results are inconsistent with a previous study [49] that found mean LOC’s response profile had some tolerance to the positions in depth defined by occlusion and disparity depth cues. The responses of subdivisions (LO1, LO2 and pFs) in the LOC were not analyzed separately in the previous study. We inferred that the inclusion of pFs in LOC may result in the inconsistent results due to the possible function segregation of LO and pFs. Moreover, the different experimental designs, the stimuli and disparity magnitudes between the two studies could also lead to the inconsistent results. Furthermore, Gilaie-Dotan et al. [15] found that activation in the ventral LOC was greater for “front” objects by random dot stereograms (RDS) using UD than “back” objects by RDS using CD. In our study, the “front/back” stimuli were generated by shapes using CD/UD. In contrast, the “front/back” objects in Gilaie-Dotan’s study were generated by changing the disparity of the background part in the RDS to UD/CD. The “front/back” stimuli corresponded to CD/UD in our study and UD/CD in Gilaie-Dotan’s study. Meanwhile, it should be noted that the stimuli used in Gilaie-Dotan’s study could produce a “concave/convex” effect, while the stimuli used in our study did not have such effect. Therefore, the different type of stimuli that were used in the two studies possibly lead to the differences of the results.

### GLM and MVPA

GLM analysis is a univariate method that can characterize the relationship between the experimental condition and individual brain voxels by examining voxels in isolation without considering the interaction among voxels. GLM can identify the voxels that show statistically significant responses to the experimental conditions [21]. In contrast, the MVPA is a multivariate approach and can decode the information that is represented by multi-voxel patterns of activity. Some studies report that GLM and MVPA analyses did identify some overlapping regions [50–52]. However, other studies found the brain areas that were revealed by GLM and MVPA were not consistent and demonstrated that MVPA might provide a different view of the functional organization of mental processing compared to GLM [53–57].

In the current study, GLM and MVPA obtained consistent results for disparity processing and inconsistent results for disparity sign processing. GLM did not detect significant differences in the dorsal visual pathway between UD and CD processing while MVPA found that the dorsal pathway showed high accuracy in the classification of UD and CD. The results may suggest the voxels that produced similar responses to different conditions still can be useful to the discrimination of the different conditions. Therefore, GLM and MVPA can be used together to provide the complementary information for each other.

## Limitation

This study only conducted the fMRI experiment with two runs. Due to the limited numbers of task runs, we used cross-validation method described in 2.6.2 instead of conventional leave one run out cross-validation. However, the small number of task runs might affect the MVPA results some unwilling noise correlation etc (contaminated in one run accidentally) may bias the trained weights of the classifier and that bias may be also reflected the final classification performance. Due to the limitation of the task runs per participant, the prediction accuracies of MVPA results were lower than those of previous study [16]. Moreover, the disparity magnitudes used in the current study are greatly different from the previous studies [16–18]. Although our main results were consistent with the previous study, there are some small differences between this study and those previous studies. Both the cross-validation procedure and the disparity magnitude possibly resulted in the differences of results.

## Conclusion

Taken together, our study used GLM and MVPA methods to define the brain regions that show selective responses and discriminative power to disparity sign, respectively. The results indicated that, the dorsal visual areas showed higher discriminative power for the disparity signs than the ventral visual areas. Moreover, the LO in the LOC of the ventral visual areas produced significantly greater responses to UD shapes than CD shapes and showed high accuracy for the classification of UD and CD.

## Abbreviations

fMRI: functional magnetic resonance imaging
LOC: lateral occipital complex
DF: dorsal foci
MVPA: multi-voxel pattern analysis
GLM: general linear model
EPI: Echo-planar imaging
TE: echo time
TR: repetition time
FOV: field of view
CD: crossed disparity
UD: uncrossed disparity
ZD: zero disparity
ROI: regions of interest
MNI: Montreal Neurological Institute
hMT+/V5: human motion complex
FDR: false discovery rate
PALS: Population-Averaged, Landmark- and Surface-based
SVM: support vector machine
LSD: least significant difference
MTG: middle temporal gyrus
MOG: middle occipital gyrus
LO2: lateral occipital areas 2
LO1: lateral occipital areas 1
IOG: inferior occipital gyrus
RDS: random dot stereograms

## References

[1] Parker AJ (2007). Binocular depth perception and the cerebral cortex. Nat Rev Neurosci.

[2] Barlow HB, Blakemore C, Pettigrew JD (1967). The neural mechanism of binocular depth discrimination. J Physiol.

[3] Nikara T, Bishop P, Pettigrew J (1968). Analysis of retinal correspondence by studying receptive fields of rinocular single units in cat striate cortex. Exp Brain Res.

[4] Pettigrew J, Nikara T, Bishop P (1968). Binocular interaction on single units in cat striate cortex: simultaneous stimulation by single moving slit with receptive fields in correspondence. Exp Brain Res.

[5] Poggio GF, Gonzalez F, Krause F (1988). Stereoscopic mechanisms in monkey visual cortex: binocular correlation and disparity selectivity. J Neurosci.

[6] Hinkle DA, Connor CE (2001). Disparity tuning in macaque area V4. NeuroReport.

[7] Burkhalter A, Van Essen DC (1986). Processing of color, form and disparity information in visual areas VP and V2 of ventral extrastriate cortex in the macaque monkey. J Neurosci.

[8] DeAngelis GC, Newsome WT (1999). Organization of disparity-selective neurons in macaque area MT. J Neurosci.

[9] Roy J-P, Komatsu H, Wurtz RH (1992). Disparity sensitivity of neurons in monkey extrastriate area MST. J Neurosci.

[10] Backus BT, Fleet DJ, Parker AJ, Heeger DJ (2001). Human cortical activity correlates with stereoscopic depth perception. J Neurophysiol.

[11] Tsao DY, Vanduffel W, Sasaki Y, Fize D, Knutsen TA, Mandeville JB, Wald LL, Dale AM, Rosen BR, Van Essen DC (2003). Stereopsis activates V3A and caudal intraparietal areas in macaques and humans. Neuron.

[12] Neri P, Bridge H, Heeger DJ (2004). Stereoscopic processing of absolute and relative disparity in human visual cortex. J Neurophysiol.

[13] Georgieva S, Peeters R, Kolster H, Todd JT, Orban GA (2009). The processing of three-dimensional shape from disparity in the human brain. J Neurosci.

[14] Ban H, Welchman AE (2015). fMRI analysis-by-synthesis reveals a dorsal hierarchy that extracts surface slant. J Neurosci.

[15] Gilaie-Dotan S, Ullman S, Kushnir T, Malach R (2002). Shape-selective stereo processing in human object-related visual areas. Hum Brain Mapp.

[16] Preston TJ, Li S, Kourtzi Z, Welchman AE (2008). Multivoxel pattern selectivity for perceptually relevant binocular disparities in the human brain. J Neurosci.

[17] Patten ML, Welchman AE (2015). fMRI activity in posterior parietal cortex relates to the perceptual use of binocular disparity for both signal-in-noise and feature difference tasks. PLoS ONE.

[18] Goncalves NR, Ban H, Sánchez-Panchuelo RM, Francis ST, Schluppeck D, Welchman AE (2015). 7 Tesla fMRI reveals systematic functional organization for binocular disparity in dorsal visual cortex. J Neurosci.

[19] Nasr S, Tootell RB: Visual field biases for near and far stimuli in disparity selective columns in human visual cortex. *NeuroImage* 2016. 10.1016/j.neuroimage.2016.09.012 PMC5346058 27622398

[20] Minini L, Parker AJ, Bridge H (2010). Neural modulation by binocular disparity greatest in human dorsal visual stream. J Neurophysiol.

[21] Friston KJ, Holmes AP, Worsley KJ, Poline JP, Frith CD, Frackowiak RSJ (1994). Statistical parametric maps in functional imaging: A general linear approach. Hum Brain Mapp.

[22] Norman KA, Polyn SM, Detre GJ, Haxby JV (2006). Beyond mind-reading: multi-voxel pattern analysis of fMRI data. Trends in cognitive sciences.

[23] Warnking J, Dojat M, Guérin-Dugué A, Delon-Martin C, Olympieff S, Richard N, Chéhikian A, Segebarth C (2002). fMRI retinotopic mapping—step by step. NeuroImage.

[24] Tootell RB, Hadjikhani N (2001). Where is ‘dorsal V4’in human visual cortex? Retinotopic, topographic and functional evidence. Cereb Cortex.

[25] Tyler CW, Likova LT, Chen C-C, Kontsevich LL, Schira MM, Wade AR (2005). Extended concepts of occipital retinotopy. Curr Med Imaging Rev.

[26] Tootell RB, Hadjikhani N, Hall EK, Marrett S, Vanduffel W, Vaughan JT, Dale AM (1998). The retinotopy of visual spatial attention. Neuron.

[27] Zeki S, Watson J, Lueck C, Friston KJ, Kennard C, Frackowiak R (1991). A direct demonstration of functional specialization in human visual cortex. J Neurosci.

[28] Kourtzi Z, Kanwisher N (2000). Cortical regions involved in perceiving object shape. J Neurosci.

[29] Kriegeskorte N, Goebel R, Bandettini P (2006). Information-based functional brain mapping. Proc Natl Acad Sci USA.

[30] Chumbley JR, Friston KJ (2009). False discovery rate revisited: FDR and topological inference using Gaussian random fields. Neuroimage.

[31] Van Essen DC, Drury HA, Dickson J, Harwell J, Hanlon D, Anderson CH (2001). An integrated software suite for surface-based analyses of cerebral cortex. J Am Med Inform Assoc.

[32] Van Essen DC (2005). A population-average, landmark-and surface-based (PALS) atlas of human cerebral cortex. Neuroimage.

[33] Press WA, Brewer AA, Dougherty RF, Wade AR, Wandell BA (2001). Visual areas and spatial summation in human visual cortex. Vision Res.

[34] Malikovic A, Amunts K, Schleicher A, Mohlberg H, Kujovic M, Palomero-Gallagher N, Eickhoff SB, Zilles K: Cytoarchitecture of the human lateral occipital cortex: mapping of two extrastriate areas hOc4la and hOc4lp. Brain Struct Funct 2015:1–21. 10.1007/s00429-015-1009-8 25687261

[35] Kujovic M, Zilles K, Malikovic A, Schleicher A, Mohlberg H, Rottschy C, Eickhoff SB, Amunts K (2013). Cytoarchitectonic mapping of the human dorsal extrastriate cortex. Brain Struct Funct.

[36] Malikovic A, Amunts K (2007). A, Mohlberg H, Eickhoff S, Wilms M, Palomero-Gallagher N, Armstrong E, Zilles K: Cytoarchitectonic analysis of the human extrastriate cortex in the region of V5/MT + : a probabilistic, stereotaxic map of area hOc5. Cereb Cortex.

[37] Talairach J, Tournoux P: Co-planar stereotaxic atlas of the human brain. 3-Dimensional proportional system: an approach to cerebral imaging. 1988.

[38] Hadjikhani N, Liu AK, Dale AM, Cavanagh P, Tootell RB (1998). Retinotopy and color sensitivity in human visual cortical area V8. Nat Neurosci.

[39] Grill-Spector K, Kourtzi Z, Kanwisher N (2001). The lateral occipital complex and its role in object recognition. Vision Res.

[40] Janssen P, Vogels R, Orban GA (1999). Macaque inferior temporal neurons are selective for disparity-defined three-dimensional shapes. Proc Natl Acad Sci.

[41] Uka T, Tanaka H, Yoshiyama K, Kato M, Fujita I (2000). Disparity selectivity of neurons in monkey inferior temporal cortex. J Neurophysiol.

[42] Tanaka H, Uka T, Yoshiyama K, Kato M, Fujita I (2001). Processing of shape defined by disparity in monkey inferior temporal cortex. J Neurophysiol.

[43] Malach R, Reppas J, Benson R, Kwong K, Jiang H, Kennedy W, Ledden P, Brady T, Rosen B, Tootell R (1995). Object-related activity revealed by functional magnetic resonance imaging in human occipital cortex. Proc Natl Acad Sci.

[44] Grill-Spector K, Kushnir T, Edelman S, Avidan G, Itzchak Y, Malach R (1999). Differential processing of objects under various viewing conditions in the human lateral occipital complex. Neuron.

[45] Larsson J, Heeger DJ (2006). Two retinotopic visual areas in human lateral occipital cortex. J Neurosci.

[46] Janssen P, Vogels R, Orban GA (2000). Selectivity for 3D shape that reveals distinct areas within macaque inferior temporal cortex. Science.

[47] Murray SO, Boyaci H, Kersten D (2006). The representation of perceived angular size in human primary visual cortex. Nat Neurosci.

[48] Weidner R, Plewan T, Chen Q, Buchner A, Weiss PH, Fink GR (2014). The moon illusion and size–distance scaling—evidence for shared neural patterns. J Cogn Neurosci.

[49] Kourtzi Z, Erb M, Grodd W, Bülthoff HH (2003). Representation of the perceived 3-D object shape in the human lateral occipital complex. Cereb Cortex.

[50] Mourão-Miranda J, Bokde AL, Born C, Hampel H, Stetter M (2005). Classifying brain states and determining the discriminating activation patterns: support vector machine on functional MRI data. NeuroImage.

[51] Willms JL, Shapiro KA, Peelen MV, Pajtas PE, Costa A, Moo LR, Caramazza A (2011). Language-invariant verb processing regions in Spanish–English bilinguals. Neuroimage.

[52] Wang Z, Childress AR, Wang J, Detre JA (2007). Support vector machine learning-based fMRI data group analysis. NeuroImage.

[53] Jimura K, Poldrack RA (2012). Analyses of regional-average activation and multivoxel pattern information tell complementary stories. Neuropsychologia.

[54] Davis T, LaRocque KF, Mumford JA, Norman KA, Wagner AD, Poldrack RA (2014). What do differences between multi-voxel and univariate analysis mean? How subject-, voxel-, and trial-level variance impact fMRI analysis. NeuroImage.

[55] Stokes M, Thompson R, Nobre AC, Duncan J (2009). Shape-specific preparatory activity mediates attention to targets in human visual cortex. Proc Natl Acad Sci.

[56] Uncapher MR, Boyd-Meredith JT, Chow TE, Rissman J, Wagner AD (2015). Goal-directed modulation of neural memory patterns: implications for fMRI-based memory detection. J Neurosci.

[57] Chiu Y-C, Esterman MS, Gmeindl L, Yantis S (2012). Tracking cognitive fluctuations with multivoxel pattern time course (MVPTC) analysis. Neuropsychologia.

